# Socioeconomic inequalities in mortality, morbidity and diabetes management for adults with type 1 diabetes: A systematic review

**DOI:** 10.1371/journal.pone.0177210

**Published:** 2017-05-10

**Authors:** Anne Scott, Duncan Chambers, Elizabeth Goyder, Alicia O’Cathain

**Affiliations:** School of Health and Related Research, University of Sheffield, Sheffield, United Kingdom; Medical Clinic, University Hospital Tuebingen, GERMANY

## Abstract

**Aims:**

To systematically review the evidence of socioeconomic inequalities for adults with type 1 diabetes in relation to mortality, morbidity and diabetes management.

**Methods:**

We carried out a systematic search across six relevant databases and included all studies reporting associations between socioeconomic indicators and mortality, morbidity, or diabetes management for adults with type 1 diabetes. Data extraction and quality assessment was undertaken for all included studies. A narrative synthesis was conducted.

**Results:**

A total of 33 studies were identified. Twelve cohort, 19 cross sectional and 2 case control studies met the inclusion criteria. Regardless of healthcare system, low socioeconomic status was associated with poorer outcomes. Following adjustments for other risk factors, socioeconomic status was a statistically significant independent predictor of mortality in 9/10 studies and morbidity in 8/10 studies for adults with type 1 diabetes. There appeared to be an association between low socioeconomic status and some aspects of diabetes management. Although only 3 of 16 studies made adjustments for confounders and other risk factors, poor diabetes management was associated with lower socioeconomic status in 3/3 of these studies.

**Conclusions:**

Low socioeconomic status is associated with higher levels of mortality and morbidity for adults with type 1 diabetes even amongst those with access to a universal healthcare system. The association between low socioeconomic status and diabetes management requires further research given the paucity of evidence and the potential for diabetes management to mitigate the adverse effects of low socioeconomic status.

## Introduction

Type 1 diabetes, formerly known as insulin-dependent diabetes mellitus (IDDM) or juvenile onset diabetes, arises because of β-cell destruction in the pancreas. Genetics and exposure to environmental factors may play an important role, however, the exact cause of type 1 diabetes is still uncertain. These cells produce a hormone, insulin, which regulates blood glucose levels. Since endogenous production of insulin is generally absent or in very small quantities, lifelong treatment with insulin is required [1]. It is estimated that 415 million people globally have diabetes and that type 1 diabetes accounts for approximately 7–12% of cases [2].

Self-care is critical to successful outcomes for individuals with type 1 diabetes and good diabetes management has been shown to minimise the risks of long-term and short-term complications [3]. However, it is postulated that inequalities in diabetes care may potentially disadvantage individuals of low socioeconomic status (SES) [4;5].The persistence of a socioeconomic health gradient in the general population is well documented and there is considerable evidence that the least well off in society have reduced life expectancy and increased morbidity compared with the affluent [6]. Despite improvements in life expectancy, inequalities in mortality are increasing [7]. For individuals with diabetes and other chronic conditions, inequalities have particular relevance since socioeconomic disparities are likely to lead to worse outcomes related to their condition, however, relatively few studies have reported the association between socioeconomic factors and mortality in type 1 diabetes relating to adults specifically.

Reviews of socioeconomic disparities in diabetes have tended to focus predominantly on type 2 diabetes [5]. Since the aetiology and treatment of type 1 and type 2 diabetes are different [2] it cannot be assumed that the impact of socioeconomic circumstances on management and outcomes would be the same in both patient groups. In addition, although socioeconomic disparities in type 1 diabetes have been identified in paediatric populations [8;9], less research has been conducted about adults with type 1 diabetes [10]. Since self-care is essential to the achievement of successful outcomes in type 1 diabetes, access to good healthcare that facilitates patient adoption of the most effective treatment regimens is also crucial. One systematic review has investigated inequalities in relation to the prevention, diagnosis, treatment, control and monitoring of type 1 diabetes [11]. However, this study covered both type 1 and type 2 diabetes and not all included studies reported results separately for the two conditions making it difficult to determine the specific associations for type 1 diabetes. Additionally the review was conducted in 2007 and eleven papers have since been published that investigate SES in relation to type 1 diabetes [12–22]. The aim of this study was to carry out a systematic review of socioeconomic inequalities in mortality, morbidity and diabetes management (including access to treatment and diabetes control) solely in relation to adults with type 1 diabetes.

## Methods

### Search strategy

We searched six databases including: Medline (accessed via OVIDSP) (1946 to the present); PsycINFO (accessed via OVIDSP) (1987 to the present); EMBASE (accessed via OVIDSP) (1974 to the present); Web of Science (1900 to the present); CINHAL (accessed via EBSCOhost) 1982 to the present); and the Cochrane Database of Systematic Reviews (1991 to the present). There is inconsistency in defining the onset of adulthood [23–27]. We have defined adults as people above 16 years in the current review to be as inclusive as possible. The majority of studies consisted of patients who were at least 18 years of age. Only 3 studies included patients who were under 18 years of age and these are identified in Table 1. The search was carried out up to the first week of May 2016 and used MeSH headings and text terms for both adults with type 1 diabetes and socioeconomic inequalities. The terms applied across all six databases were: type 1 diabet*; insulin dependent diabet*; socioeconomic; socio-economic; social class*; social status, poverty, impoverished, inequit*, equity, access*; healthcare disparit*; health care disparit* and health status disparit*. An example of the search strategy applied to one of the databases is described in S1 Table. One reviewer (AS) searched the reference lists of papers in the final selection to identify further studies and carried out a forward citation search using a ‘snowballing’ technique [28] in order to check for more recent studies. Handsearching of the following journals focusing on diabetes research was undertaken. This included Diabetic Medicine, Diabetes Care, Diabetologia; Diabetes and Practical Diabetes for the most recent period (May 2015-May2016) to capture citations not yet added to the databases.

**Table 1:** Characteristics of included studies.

Study	Year	Country/Area	Study design	N (type 1)	Study scope
Anderson* [14]	2014	UK	Cross sectional	1621	Morbidity
Butalia [15]	2013	Canada	Cross sectional	1994	Morbidity
Chaturvedi [31]	1996	Pan-European	Cross sectional	2387	Morbidity Diabetes management
Forssas [13]	2010	Finland	Cohort	1 407 025 person years 59 917 deaths	Mortality
Forssas [32]	2003	Finland	Cohort	546 000 person years† 24 662 deaths†	Mortality
Forssas [12]	2012	Finland	Cohort	528 734 person years 18 841 deaths	Mortality
Gnavi [33]	2004	Italy	Cohort	31 264 (1608)	Mortality
Harris [34]	1993	USA	Cross sectional	2392 (124)	Diabetes management
Hepburn [35]	1994	Scotland	Cross sectional	121	Diabetes management
Johansen [36]	1986	Denmark	Cross sectional	57	Diabetes management
Karter [37]	2000	USA	Cross sectional	44 181 (2818)	Diabetes management
Leese [38]	2003	Scotland	Cohort	160 (69)	Morbidity
Lievre* [39]	2005	France	Cross sectional	2253	Morbidity
Lloyd [40]	1993	USA	Cross sectional	592	Diabetes management
Matsushima [41]	1996	Japan	Case control	180	Mortality
Mühlhauser [42]	1998	Germany	Cohort	684	Morbidity
Mühlhauser [43]	1998	Germany	Cross sectional	684	Morbidity Diabetes management
Mühlhauser [44]	2000	Germany	Cohort	3674	Mortality, Morbidity
Nadas 2009 [16]	2009	Hungary	Cross sectional	437	Morbidity Diabetes management
Osan [17]	2016	Australia	Cross sectional	93	Diabetes management
Pederson- Bjergaard [45]	2004	Denmark/UK	Cross sectional	1076	Morbidity
Perros [46]	1998	Scotland	Cross sectional	60	Diabetes management
Rawshani [18]	2015	Sweden	Cohort	24 947	Mortality, Morbidity
Robinson [47]	1984	UK	Case control	329 (95)	Morbidity
Robinson* [48]	1998	UK	Cohort	2104 (798)	Mortality
Rossing [49]	1996	Denmark	Cohort	939	Mortality
Sastre* [19]	2012	Spain	Cross sectional	1465	Morbidity Diabetes management
Secrest [20]	2011	USA	Cohort	317	Mortality
Secrest [21]	2011	USA	Cohort	317	Morbidity Diabetes management
Simmons [10]	2013	USA	Cross sectional	1894	Diabetes management
Unwin [50]	1996	UK	Cross sectional	1246 (296)	Morbidity Diabetes management
Weinstock [22]	2013	USA	Cross sectional	7012	Morbidity
Zgibor [51]	2000	USA	Cross sectional	429	Diabetes management

*Studies which included young adults (above 16 or 17 years of age)

^†^ Comparison with non-diabetes population (28 900 000 person years and 214 041 deaths)

### Study screening and selection—Inclusion and exclusion criteria

Database searches were performed by the first author. The search strategy presented in S1 Table (used for Medline) was modified as necessary for the other databases. Following the removal of duplicates, search results were initially screened on the basis of title and abstract and irrelevant papers were removed. Full papers were obtained and then screened by the first and second author who reached agreement on the studies to be included in the final review. Any disagreements about the inclusion of papers were resolved through discussion with the other authors.

Studies were included if they reported socioeconomic findings in adults (16 years and above) with type 1 diabetes in any of the following categories: mortality; morbidity arising from diabetes (short- and long-term complications); glycaemic control; insulin regimens; and access to care. Papers were included if they reported either individual SES (education, income or social status) or deprivation at group level. For practical purposes the search was limited to papers published in the English language only. In order that studies were comparable, papers were limited to countries belonging to the Organisation for Economic Co-operation and Development (OECD). All study designs were included. Primary research studies reported in peer review journals were included.

Studies were excluded if they: focused on pregnant women only (to exclude potential cases of gestational diabetes); involved only children and adolescents (below the age of 16); focused on the transition of children from paediatric to adult service; focused on type 1 and type 2 diabetes but did not report on the former separately; or if they included both adults and children but did not report on the former separately. Conference abstracts were excluded because of the difficulty in comprehensively assessing the risk of bias.

### Data extraction and quality assessment

Following the final selection of papers, the first author extracted data using a standardised checklist including: study aims and design; location; participants (sex, age, numbers and ethnicity); inclusion and exclusion criteria; clinical and non-clinical variables; analysis used; and results. Assessment of study quality, including risk of bias, was undertaken on full papers based on the Critical Appraisal Skills Programme (CASP) [29] (S2 Table) using a form adapted by the author (AS). The results of the data extraction and quality assessment were validated by the second author checking a random selection of papers. Studies were not excluded based on quality because weaknesses are likely to be present in any study and quality assessment must not become a pursuit of unattainable perfection [28]. Instead study quality was considered in the narrative review.

The guidelines encompassed in the Preferred Reporting Items for Systematic Review and Meta-analysis (PRISMA) [30] checklist (S3 Table) were used to report the findings of the systematic review.

## Results

### Study selection

**Fig 1:**
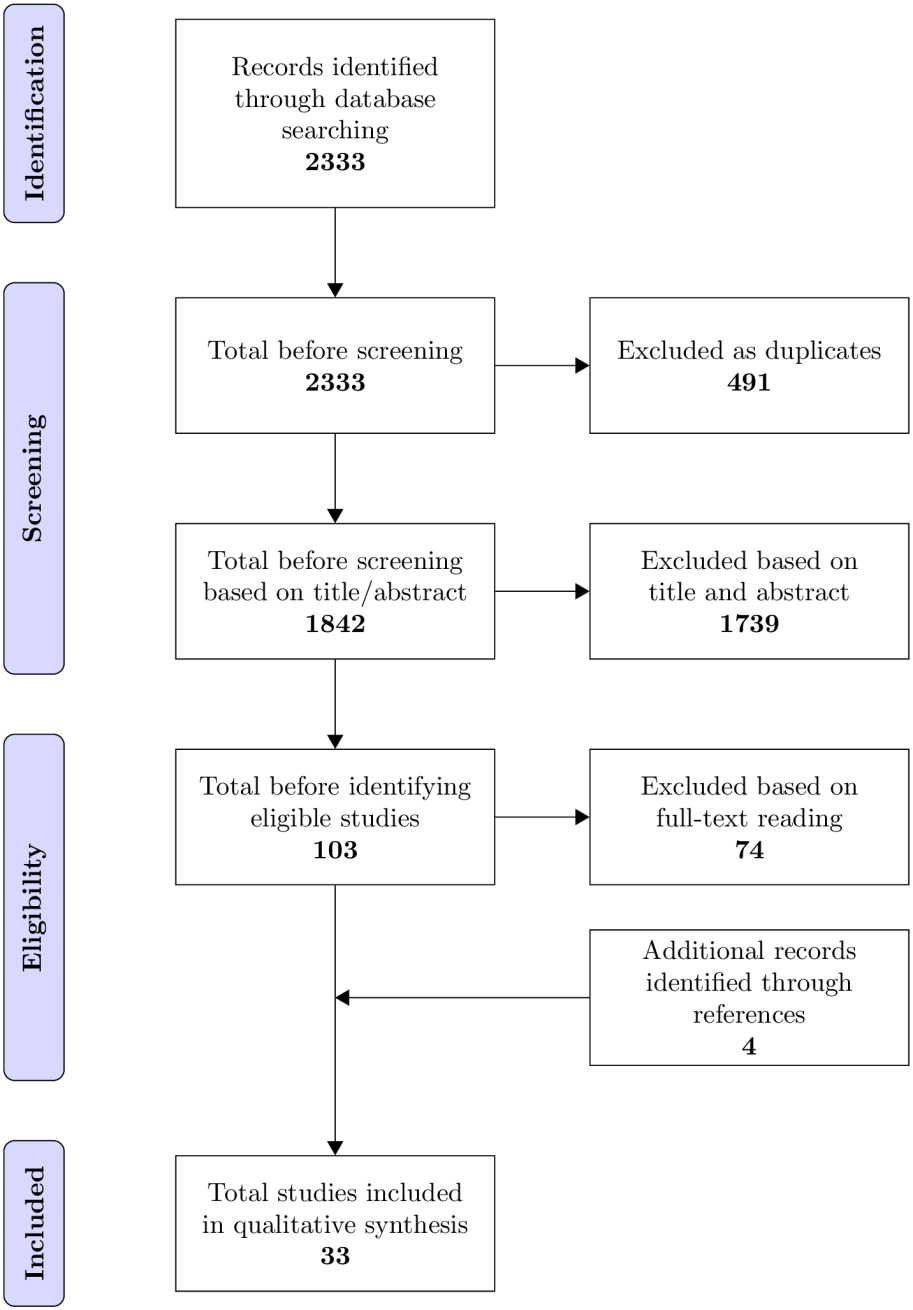
PRISMA flow diagram showing included and excluded studies.

The result of the systematic literature search is summarised in the PRISMA flow diagram (Fig 1) [30]. Following the database search 2,333 references (excluding duplicates) were retrieved. Of these 1,739 were excluded on the basis of title or abstract. We screened 103 full papers. Of these 74 were rejected including one systematic review. References from this paper were cross referenced against papers found in our own review. The references of all 29 papers found were scanned for further relevant articles. An additional 4 studies were found which were then screened by abstract and full paper reading bringing the total to 33. Hand searching of journals yielded no additional papers. The most common reasons for excluding papers following a full paper review was: type 1 diabetes and SES not covered (15 papers); type 1 diabetes and SES not reported separately for studies that included type 2 diabetes (22 papers); results for adults not reported separately for studies that included children (10 papers) or age range not specified or outside study scope (13 papers).

### Characteristics and quality of studies

The characteristics of the 33 studies are summarised in Table 1. The majority were from Europe (22 studies) with 8 carried out in the USA and one study each conducted in Japan, Australia and Canada. Study designs utilised were mainly cohort (12) or cross sectional (19). The majority of studies assessed SES at the individual level. The most commonly used measures were social group (based on occupation) and education. Four studies measured SES using deprivation at area level. Two studies used the Townsend score, one study used the Carstairs index and one study used the Index of Relative Disadvantage (Australian Bureau of Statistics).

Three main factors limiting the quality of some studies were identified (S3 Table). First, some studies utilised subjective outcome measures based on self-reporting by participants and hence may have been subject to reporting bias. Second, 10 studies did not take account of confounding factors during analysis. In total, 23 of 33 studies adjusted for confounding factors including age, sex, and diabetes duration as well as known risk factors including glycaemic, lipid and blood pressure control. Third, the results of some studies were potentially affected by selection bias involving underrepresentation of adults of low SES.

Associations between SES and mortality were assessed in 10 studies; associations between SES and morbidity and SES and diabetes management 16 studies each. Some studies considered more than one of these categories. Due to the variation in defining SES variables across studies it was not possible to carry out a meta-analysis. Instead, a narrative synthesis was undertaken.

### Studies reporting all-cause mortality—characteristics

In total, 10 papers investigating mortality were eligible for inclusion in this review. Of these, 6 studies were population based and 4 studies recruited patients at specialist diabetes clinics in secondary care (centre based). Studies were carried out in the UK, Finland, Sweden, Italy, Germany, Denmark, Japan and the USA (Table 2).

**Table 2:** Mortality in adults with type 1 diabetes.

Study	Mortality assessment (cohort follow-up)	Methods to assess SES	SES variable(s)	Key findings for adults with type 1 diabetes
Forssas *et al*. [32]	Population based (1981–1985 1991–1996)	Social class	✔^a^	*Adjusted analysis*: A socioeconomic gradient in mortality was found for individuals with type 1 diabetes. For men with type 1 diabetes, circulatory diseases and diabetes contributed 48% and 34% respectively of the mortality disparities between blue and white collar workers. For women 42% of the socioeconomic gradient was due to diabetes.
Forssas *et al*. [13]	Population based (1991–2003)	Social class	✔^a^	*Adjusted analysis*: In the period 1995–2003 a socioeconomic mortality gradient was found for individuals with type 1 diabetes. Disparities in mortality between manual workers versus non-manual workers were greatest in relation to alcohol related deaths for men (RR: 1.97 (1.51–2.57)) and women (RR 2.13 (1.17–3.86)).
Forssas *et al*. [12]	Population (2000–2003)	Social class Education Income Employment Municipality	✔^a^^,^ ✔^b^ ✔^c^ ✔^e^ x^f^	*Unadjusted and adjusted analysis*: Socioeconomic differences in mortality related to occupation, education, and income. Among men with type 1 diabetes mortality differences were largest for the long-term unemployed (aged 30–64, RR 3.85 (3.00–4.94) compared with employed and for low versus high income RR 1.96 (1.78–2.17). For women mortality differences were largest for unemployment RR 3.32 (1.88–5.88) and education (RR 2.35 (1.84–3.00)). No significant mortality differences were found for type of municipality of residence.
Gnavi *et al*. [33]	Population based (1991–1999)	Education	✔^b^	*Adjusted analysis*: Individuals with primary school or no formal education were three to four times as likely to die during the study period as those with higher educational level (For men: HR = 3.1, 95% CI: 1.6–6.1; For women: HR = 4.4, 95% CI: 1.6–12.3).
Matsushima *et al*. [41]	Population based	Education Income	✔^b^ x ^c^	*Unadjusted analysis*: Deceased cases were more likely to have lower educational attainment (RR 2.5 CI 0.9–7.2). No statistically significant association was found in relation to income. *Adjusted analysis*: In two of the three models the association between lower educational attainment and deceased cases remained. After adjustment for complications (chronic and acute) education no longer reached significance as an independent variable.
Mühlhauser *et al*. [44]	Diabetes centre (1996–1998)	Social status	✔^d^	*Adjusted analysis*: Low social status was a significant predictor for mortality (HR 1.4, 95% CI 1.1–1.8, p<0.0037). Other predictors included: nephropathy, smoking, serum cholesterol, age, male sex and systolic blood pressure.
Rawshani *et al*. [18]	Population based (2006–2008 mean (SD) follow-up of 6.0 (1.0) years	Education Income	✔^b^ ✔^c^^,^	*Adjusted analysis*: Cox adjusted survival curves for death indicated that income and education were significantly associated with survival (p <0.05). Risk of death was three times greater for those in the two lowest income quintiles compared to the highest income quintile. Risk of cardiovascular death and diabetes-related death was three times as much and the risk of diabetes-related death was twice as much for these quintiles following adjustments in the model. Those with higher levels of education had lower risk of death than those with ≤ 9 years of education. These results were weakened in the maximally adjusted model although risk of fatal stroke remained significant.
Robinson *et al*. [48]	Diabetes centre (Mean (SD) follow up of 8.4 (0.9) years.	Social class Education Employment	x^a^ ✔^b^ ✔^e^	*Adjusted analysis*: After adjusting for duration of diabetes mortality rates for were significantly higher for those who left school before 16 years compared to those who left at 16 or later (adjusted OR 4.0, CI 1.96–8.06, p<0.05). Mortality rates were approximately three times higher in the unemployed compared with those who were employed (adjusted OR 3.10, CI 1.67–5.79, p<0.001). After adjusting for age employment status was no longer predictive of mortality.
Rossing *et al*. [49]	Diabetes centre (1984–1985)	Social class	✔^a^	*Adjusted analysis*: Low socioeconomic group was significantly associated with increased mortality. Social class V versus social class IV RR 1.70 95% CI 1.25–2.31, p <0.001.
Secrest *et al*. [20]	Diabetes centre (median follow-up time of 16.3 years, range 2.0–21.7 years)	Social class Education Income	x^a^ ✔^b^ ✔^c^^,^	*Unadjusted analysis*: Individuals in the highest income and education group had similar mortality rates to local general population. Individuals with lower income and education had rates of mortality that were five times higher than the general population. Individuals without a college degree were three times more likely to die than those without a college degree HR 3.0, 95% CI 1.2–7.8, p = 0.02. *Adjusted analysis*: The relationship between education and mortality was attenuated (HR reduced from 3.0 to 2.1) after adjusting for confounders and risk factors. Income was largely unaffected by adjustments (HR = 3.2 reduced to 3.0).

^a^ Social class (occupation),

^b^ Education,

^c^ Income,

^d^ Aggregate score of occupation and education,

^e^ Employment,

^f^ Municipality.

CI, confidence interval; HR, hazard ratio; RR, relative risk; OR, odds ratio; SD, standard deviation.

### Social gradient in adults with type 1 diabetes compared with the general population

In four studies the social gradient in mortality for adults with type 1 diabetes was steeper compared with the general population. In Italy the risk of death during the study period was estimated to be double (for men) and triple (for women) [33] and in the USA the risk of death was four times higher [20]. In Finland comparisons of mortality between adults with type 1 diabetes and the general population revealed an increase in socioeconomic disparities over time [32].

### Association between SES and mortality in the type 1 diabetes adult population

In the 10 papers reporting mortality in adults with type 1 diabetes, every paper, regardless of design, country, or SES measure reported an association between higher mortality and lower SES in either unadjusted or adjusted analyses (Table 2). All of these countries except the USA have a form of universal healthcare system. In 5 of 6 studies lower SES measured by occupation was significantly associated with higher levels of mortality [12;13;20;32;49]. In all 6 studies using education as a measure of SES, lower levels of education were found to be associated with higher mortality [12;18;20;33;41;48]. Higher mortality was significantly associated with lower levels of income in 3 of 4 studies [12;18;20]. In one study lower social status (measured by an aggregate of both occupation and education) was a significant predictor of mortality [44]. In two studies investigating the variable, unemployment was found to be a significant predictor of mortality. In a Finnish study, the largest relative differences in all-cause mortality were found amongst men and women who were unemployed versus those who were employed [12]. Similar results were found in a UK study where mortality rates of unemployed individuals were approximately three times higher than those who were employed [48].

### Mortality and confounding factors

Although an association with SES was found in unadjusted analyses for each study investigating mortality, when other known risk factors were modelled using adjusted analysis a more complex picture was revealed. Higher rates of mortality were associated with at least one SES measure in 9 of 10 studies [12;13;18;20;32;33;44;48;49]. Occupation and/or social status was found to be an independent predictor of mortality in 5 studies [12;13;32;44;49] whereas associations between education and mortality were less consistent. In 3 studies a strong association between education and mortality was lessened when other risk factors (HbA1c, cholesterol, hypertension and microalbuminuria) known to predict mortality in type 1 diabetes were taken into account [18;20;41]. In contrast education remained as an independent predictor of mortality in 3 studies [12;33;48]. In adjusted analysis income was associated with mortality in 3 of 4 studies [12;18;20]. For example, in a Swedish study (in a maximally adjusted model), the risk of all-cause mortality and diabetes-related death for individuals in the two lowest income quintiles compared with those in the highest income quintile was twice as great and the risk of cardiovascular death was three times as much. In two of the studies income was a stronger predictor of mortality than education [18;20].

These results demonstrate the difficulty in identifying consistent independent predictors of mortality, since variables may be associated with one another [20]. For example, although cardiovascular mortality was consistently found to be a major cause of death, not all studies explored lifestyle factors such as smoking and alcohol that may act as confounding factors. Nevertheless, a relationship between social status and mortality persisted in one study even when traditional risk factors for cardiovascular disease were taken into account [44] and in the largest sample of adults with type 1 diabetes in this review, low SES increased the risk of death by 2–3 even when adjustments were made for confounding factors [18].

### Studies reporting morbidity—characteristics

Sixteen studies met the inclusion criteria for morbidity and SES in adults with type 1 diabetes. Thirteen studies were conducted in Europe. EURODIAB [31], one of the largest studies of diabetes complications within the review included 31 clinics in 16 countries. Two of 31 clinics were outside the OECD (Romania and Croatia). One study was conducted in Canada and two in the USA. Designs included cohort and cross sectional studies. A variety of measures were used to determine SES. The majority of authors chose individual measures such as education, income or occupation. Some used an aggregate score comprising two or three individual measures. Three studies used an area level measure based on indices of deprivation (such as the Carstairs index [52] or the Townsend score [53]). The findings for morbidity are summarised in Table 3. The studies investigated long-term complications (microvascular and macrovascular) and short-term complications (hypoglycaemia and ketoacidosis) associated with diabetes. Some studies investigated both long- and short-term complications.

**Table 3:** Morbidity (short- and long-term complications associated with type 1 diabetes).

Study	Morbidity measures	Methods to assess SES	SES variable(s)	Key findings for adults with type 1 diabetes
Anderson *et al*. [14]	Painful neuropathy	Deprivation	**✔** ^g^	*Adjusted analysis*: Each unit increase in deprivation score was associated with an increased risk of being prescribed medication for neuropathic pain (OR 1.11 (1.05–1.17), p <0.001).
Butalia *et al*. [15]	Ketoacidosis	Education* Income*	x ^b^ x ^c^	*Unadjusted and adjusted analysis*: No association was found between hospitalisation for diabetic ketoacidosis (DKA) and either income or education.
Chaturvedi *et al*. [31]	Micro and macrovascular complications	Social class Education	**✔**^a^ **✔**^b^	*Unadjusted and adjusted analysis*: Significantly lower rates of microalbuminuria and proliferative retinopathy were found for college educated men versus primary educated men. College education people were less likely to be smokers (p <0001).
Leese *et al*. [38]	Hypoglycaemia	Deprivation	**✔** ^g^	*Unadjusted analysis*: Increasing deprivation was associated with severe hypoglycaemia (p <001).
Lievre *et al*. [39]	Complications, ketoacidosis, hypoglycaemia	Social class	**✔**^f^	*Unadjusted analysis*: Risk of having at least one complication was increased for each decrease in SES score. DKA was associated with SES score. Hypoglycaemia was not associated with SES.
Mühlhauser *et al*. [42]	Hypoglycaemia	Social class	**✔**^e^	*Adjusted analysis*: Low SES was a statistically significant predictor of severe hypoglycaemia.
Mühlhauser *et al*. [43]	CVD risk factors, nephropathy	Social class	**✔**^e^	*Unadjusted analysis*: Fewer adults of higher social status had either macrovascular complications or foot complications. An association between high SES and less likelihood of smoking was statistically significant. *Adjusted analysis*: High SES was associated with lower risk of nephropathy.
Mühlhauser *et al*. [44]	Microvascular complications	Social class	**✔**^d^	*Adjusted analysis*: Low SES was significantly predictive of complications (a combination of blindness or amputations or renal replacement therapy) even after adjusting for other known risk factors.
Nadas *et al*. [16]	Cardiometabolic risk factors	Education	**✔**^b^	*Unadjusted analysis*: Higher prevalence of metabolic syndrome and smoking were both associated with low versus high education.
Pederson- Bjergaard *et al*. [45]	Hypoglycaemia	Education	**✔**^b^	*Unadjusted analysis*: Primary school education was associated with a higher rate of hypoglycaemia. *Adjusted analysis* education was no longer associated with severe hypoglycaemia.
Rawshani *et al*. [18]	CVD events Non-fatal CHD	Education Income	**✔**^b^ **✔**^c^	*Adjusted analysis*: Individuals in the two lowest income quintiles had a two to three times higher risk of CVD events than those in the highest income quintile. Compared with low educational level having a high education was associated with approximately 30% lower risk of stroke.
Robinson *et al*. [47]	Risk factors for micro and macrovascular complications	Social class	**✔**^a^	*Unadjusted analysis*: Differences between low and high SES were only observed amongst women with the latter smoking less and having a lower mean triglyceride level than the former.
Sastre *et al*. [19]	CVD risk factors	Education	**✔**^b^	*Unadjusted analysis*: Low educational level or no primary education was associated with a greater prevalence of risk factors.
Secrest *et al*. [21]	Risk of complications including CHD events	Social class Education Income	**✔**^a^ **✔**^b^ **✔**^c^	*Unadjusted analysis*: All complications (ESRD, CHD, LEAD and AN) were associated with at least one SES measure. *Adjusted analysis*: Following adjustments for other variables (including clinical risk factors) only the association between LEAD and income remained. Peripheral retinopathy was not associated with SES in either unadjusted or adjusted analysis. Smoking status was less common in high SES categories.
Unwin *et al*. [50]	CVD risk factors	Deprivation	**✔**^g^	*Unadjusted analysis*: Increasing deprivation was significantly associated with mean serum cholesterol and smoking.
Weinstock *et al*. [22]	Ketoacidosis, hypoglycaemia	Education Income	**✔**^b^ **✔**^c^	*Adjusted analysis*: Low SES (education and income) was associated with higher frequency in both DKA and severe hypoglycaemia.

^a^ Social class (occupation),

^b^ Education,

^c^ Income,

^d^ Aggregate score of occupation and education,

^e^ Aggregate score of occupation, education and income,

^f^ Aggregate score of employment status, occupation, education and living alone,

^g^ Area Deprivation score..

OR, odds ratio; CVD, cardiovascular disease; CHD, coronary heart disease; ESRD, end-stage renal disease; LEAD, lower-extremity artery disease; AN, autonomic neuropathy.

*Measured at neighbourhood level.

### Association between SES and morbidity

Of the 16 primary studies, 15 found a significant association with at least one measure of SES for long- or short-term complications associated with diabetes (Table 3). Eleven of the studies reporting an association between low SES and poorer outcomes were conducted in countries with a universal healthcare system.

#### Long-term complications

All 8 studies reporting on SES and risk factors for cardiovascular disease (CVD) or CVD events found an increased risk associated with low SES [16;18;19;21;31;43;47;50]. An association between low SES and increased rates of complications arising from diabetes was observed in 5 studies [21;31;39;43;44]. Complications investigated included: renal disease [21;39;43;44]; proliferative retinopathy [31;39;43]; blindness [44]; lower extremity arterial disease [21;39;43]; painful neuropathy [14] and autonomic neuropathy [21].

#### Short-term complications

In unadjusted analysis an association between SES and severe hypoglycaemia was found in 4 studies [22;38;42;45] whereas one study found no association [39]. In 2 of 3 studies ketoacidosis was associated with SES [22;39]. One study (conducted in Canada) did not find an association between ketoacidosis and low SES [15]. The authors suggested that this may have been as a result of using group level data rather than individual measures of income and education. A possible explanation was their acknowledgment that Calgary is a relatively affluent city and that their study lacked socioeconomic diversity. Although the study by Lievre et al. [39] found no association between hypoglycaemia and SES, the authors acknowledged that the least well off patients were under-represented in their study.

### Morbidity and confounding factors

Of the 10 studies utilising adjusted analysis, 8 found that low SES was an independent predictor of either short- or long-term complications associated with diabetes. Six studies found SES was an independent predictor of long-term complications arising from type 1 diabetes [14;18;21;31;43;44]. Following adjustments for other risk factors including age, diabetes duration, sex, and BMI, low SES was significantly associated with severe hypoglycaemia in one study [42] and both severe hypoglycaemia and ketoacidosis in another study [22]. Two studies found no association between SES and hypoglycaemia [45] or ketoacidosis [15] following adjusted analysis.

### Studies reporting diabetes management—characteristics

Sixteen studies were found that investigated SES and at least one aspect of diabetes management (glycaemic control, self-monitoring of blood glucose levels (SMBG), insulin regimens or access to specialist diabetes care). The studies were carried out in the USA (5), UK (3), Australia (1) and other European countries (7). Findings are summarised in Table 4. Five of these studies were also included in the morbidity review [16;21;31;43;50]. All except one study were cross sectional in design. Twelve studies investigated glycaemic control whereas few studies were found that investigated SES in relation to SMBG, insulin regimens or access to specialist healthcare in relation to adults with type 1 diabetes.

**Table 4:** Diabetes management (glycaemic control, self-monitoring of blood glucose levels (SMBG), access to specialist care (SC) and adoption of an intensive insulin regimen).

Study	DM Measures	Methods to assess SES	SES variable(s)	Key findings for adults with type 1 diabetes
Chaturvedi *et al*. [31]	Glycaemic control Access to SC	Education	✔^b^	*Unadjusted analysis*: Mean percentage HbA1c was worse in primary educated versus college educated men and women. For men and women attendance at specialist diabetes services was associated with education level (p = 0.003 and p <0.0001 for trend respectively).
Harris *et al*. [34]	SMBG	Education Income	x^b^ x^c^	*Unadjusted analysis*: Education and income were not associated SMBG.
Hepburn *et al*. [35]	Glycaemic control	Social class Education	x^a^ x^b^	*Unadjusted analysis*: No association between glycaemic control and either social class or education.
Johansen *et al*. [36]	Glycaemic control	Social class	**✔**^a^	*Unadjusted analysis*: Significantly more patients with HbA1c <8.5% were in social classes 1–3 compared with social classes 4–5 (p = 0.0025). HbA1c was lower in social class 1–2 compared with social class 5 (median HbA1c 8.1% versus 10.3% p = 0.02).
Karter *et al*. [37]	SMBG	Education Income	x^b^ **✔**^d^	*Adjusted analysis*: Low neighbourhood income levels (< $13,959 average annual income) were predictive of SMBG being carried out less frequently than recommended.
Lievre *et al*. [39]	Diabetes management score	Social class	x^f^	*Unadjusted analysis*: A non-significant trend was found between SES and a diabetes management score that included HbA1c, insulin regimen, blood pressure and attendance at specialist care (p = 0.053).
Lloyd *et al*. [40]	Glycaemic control	Education Income	**✔**^a^✔^b^	*Unadjusted analysis*: Low income and a lower level of education were significantly associated with poorer glycaemic control (income p < 0.05 and education p < 0.001). *Adjusted analysis*: Level of *e*ducation remained a significant independent predictor of glycaemic control (p = 0.0008).
Mühlhauser *et al*. [43]	Glycaemic control SMBG Access to SC Adoption of an IIR	Social class	**✔**^g^	*Unadjusted analysis*: Lower social class was associated with higher HbA1c values (p < 0.0001) and less frequent SMBG. A higher percentage of higher SES attended specialist diabetes services (OR = 1.36, CI: 1.17–1.56 p <0.0001). There was a strong association between lower social class and the intensity of insulin regimen (p < 0.0001). Insulin adjustment was carried out more by high SES than low SES participants (p < 0.0001).
Nadas *et al*.[16]	Glycaemic control	Education	✔^b^	*Unadjusted analysis*: Glycaemic control was worse in low education patients versus high (HbA1c 8.8±1.6 versus 7.9±1.4% p = 0.0006).
Osan *et al*. [17]	Glycaemic control	Deprivation	x^e^	*Unadjusted analysis*: SES was not associated with glycaemic control.
Perros *et al*. [46]	Adoption of an IIR	Social class Education	**✔**^a^✔^b^	*Unadjusted analysis*: Basal bolus regimens were associated with higher levels of education (p = 0.03) and higher social class (p = 0.002).
Sastre *et al*. [19]	Glycaemic control	Education	**✔**^b^	*Unadjusted analysis*: Glycaemic control (HbA1c ≤ 7%) was associated with educational level (middle plus higher education versus primary or no education). *Adjusted analysis*: Better glycaemic control was associated with secondary or higher education (p<0.05).
Secrest *et al*. [21]	Glycaemic control Adoption of an IIR	Social class Education Income	**✔**^a^ **✔**^b^ **✔**^c^	*Unadjusted analysis*: HbA1c decreased with increased income level (p = 0.01). College graduates compared with individuals with less education were more likely to be on an IIR (23.7% versus 12.8% p ≤ 0.05). Individuals in professional occupations versus non-professional occupation were more likely to be on an IIR (27.2% versus 8.7%, p ≤ 0.01).
Simmons *et al*. [10]	Glycaemic control	Income Education Employment	**✔**^b^✔^c^ x^h^	*Unadjusted analysis*: Participants with excellent control compared with fair/poor control were more likely to have a higher income (p <0.0001), higher education level p <0.0001).
Unwin *et al*. [50]	Glycaemic control	Deprivation	x^e^	*Unadjusted analysis*: No significant association between HbA1c and SES.
Zgibor *et al*. [51]	Glycaemic control Access to SC	Education Income	**✔**^b^ **✔**^c^	*Unadjusted analysis*: Higher levels of HbA1c were significantly associated with lower levels of income and lower levels of education (p ≤0.05 for both). Individuals accessing specialist care were significantly more likely to have higher education levels and to have income above $20,000 (OR 2.1 (1.4–3.2, p ≤0.001 and OR 1.8 (1.1–3.0, p ≤0.005) respectively.

^a^ Social class

^b^ Education

^c^ Income

^d^ Neighbourhood income,

^e^ Area Deprivation score,

^f^ Aggregate score of employment status, occupation, education and living alone,

^g^ Aggregate score of occupation, education and income,

^h^ Employment.

OR, odds ratio.

### Association between SES and diabetes management

In 11 of 16 studies an association between an aspect of diabetes management and SES was found. Six of the studies reporting an association were conducted in countries with a universal healthcare system. One study scoring a combination of factors relating to ideal disease management (see Table 4) found a non-significant trend with the score and SES. The authors acknowledged that selection bias (the lack of individuals from low SES in the sample) and the universal health care system in France may have accounted for the lack of statistical significance [39].

#### Glycaemic control

Twelve studies explored associations between HbA1c levels and SES (Table 4). An association between low SES (as measured by education, social class or income) and poorer glycaemic control was found in nine of the twelve studies [10;16;19;21;31;36;40;43;51]. Two studies found no SES association with glycaemic control [17;35].

#### Self-monitoring of blood glucose (SMBG) levels

The evidence for socioeconomic association with SMBG in adults with type 1 diabetes was weak. Only three studies were found that met the inclusion criteria. All three of these studies were based on self-reporting which may be an unreliable measure [54]. Harris et al. [34] found no association between SES and SMBG whereas a German study and a study conducted in the USA found an association between low SES and lower rates of SMBG [37;43].

#### Access to specialist diabetes services

All three studies included in the review of access to diabetes care found an association between attendance at specialist diabetes centres and SES for adults with type 1 diabetes [31;43;51]. In the EURODIAB study, Chaturvedi et al. [31] postulated that access to care may be a possible explanation for poorer outcomes for low socioeconomic groups. The authors assessed attendance at the clinic using the proxy of last available HbA1c result (over two years). A social gradient for attendance at the clinic according to educational level was found. For men and women with primary level education, 67% had at least one reported HbA1c result in the previous two years compared with 79% (men) and 82% (women) with college education.

In a study conducted in the USA, Zgibor et al. [51] found that attending specialist adult type 1 diabetes services was associated with education and income. Additionally, those individuals attending specialist diabetes services were more likely: to have received diabetes education recently; to be knowledgeable about HbA1c; to have carried out SMBG; and to be injecting insulin more than twice daily. Those individuals with a lower HbA1c were more likely to have attended specialist diabetes services. These findings were similar in a population with a universal healthcare system. In a German study, Mühlhauser et al. [43] found that more of the individuals in the higher socioeconomic group compared with the lower socioeconomic group consulted diabetes clinics. However, more patients of low SES compared with high SES reported visiting a primary care physician during the previous year.

#### Adoption of an intensive insulin regimen

Associations between intensity of insulin regimens and SES were found in three studies [21;43;46]. For example, individuals in higher socioeconomic groups injected insulin more frequently each day and carried out more insulin adjustments. They were also more likely to be using insulin pumps, were better informed and a higher percentage had attended structured education than individuals in lower socioeconomic groups [43]. A strong association was found between SES and daily injections, with fewer of the lower SES patients on intensified regimens. The authors concluded that since the same opportunities had been provided for all socioeconomic groups, equality of access was demonstrated in their study and inequalities in uptake of services were viewed as stemming from a deficit in health motivation amongst people from lower socioeconomic groups [43].

A study conducted in Scotland found that individuals on a basal bolus regimen compared with twice daily regimens tended to be younger, to adjust their regimens more frequently, to be more highly educated and were of a higher SES [46]. In a study conducted in the USA adults with more education or better employment were significantly more likely to be on intensive regimens by age 28 [21].

Insulin pumps represent the most intensive of all regimens in type 1 diabetes since insulin is continuously administered subcutaneously via the equipment. Insulin pump therapy was mentioned in one of the three studies investigating intensity of regimen and associations with SES [43]. However, this form of therapy was not reported separately.

### Diabetes management and confounding factors

Only 3 of 16 studies carried out analysis to determine if SES was an independent predictor. For example, only 2 of 12 studies investigating glycaemic control utilised adjusted analysis [19;40], however, both studies found that SES was independently associated with glycaemic control. In one of these studies conducted in the USA, multiple regression demonstrated that level of education was an independent predictor of glycaemic control (p = 0.0008) [40]. In Spain, a study using adjusted (logistic regression) analysis found that better glycaemic control was also independently associated with higher levels of education (p <0.05) [19]. Only 1 of 3 studies investigating SMBG used adjusted analysis. This study conducted in the USA found that low neighbourhood income levels were predictive of SMBG being carried out less frequently than recommended (p < 0.05) [37]. None of the studies investigating intensity of regimens or access to specialist diabetes care carried out adjusted analysis, hence there was a lack of evidence confirming SES as an independent predictor in these papers.

## Discussion

### Summary of findings

This review has identified socioeconomic inequalities in relation to health outcomes and diabetes management for adults with type 1 diabetes in three key areas. First, it appears that social status, education, deprivation and unemployment are all associated with mortality. The association of SES factors with mortality in type 1 diabetes is in contrast to the lack of a consistent social gradient noted in a study in which both type 1 and type 2 diabetes were reported together [32]. Second, low SES is associated with morbidity arising from diabetes including both long- and short-term complications. SES associations with both long- or short-term complications for adults with type 1 diabetes have not been investigated separately from type 2 diabetes in a systematic review. Third, in relation to factors which may lessen the likelihood of complications, for example having good control of blood glucose levels, low SES consistently predicts poor glycaemic control. These findings resonate with previous reviews of inequalities in general diabetes management that show that individuals of low SES have poorer control of their condition than their counterparts in high SES groups (for example the former have HbA1c values above the recommended levels [5;11]). The current review identifies that these inequalities relate not only to individuals with type 2 diabetes but also to adults with type 1 diabetes.

In addition, it appears that those of low SES are less likely to adopt intensive insulin regimens. This is important because more intensive regimens are likely to result in better outcomes [3]. Although previous studies of children with type 1 diabetes in the USA have shown that low SES is associated with disparities in the uptake of insulin pumps [8], there is a paucity of research in relation to the uptake of this therapy amongst adults of low SES, particularly in countries providing universal healthcare.

Overall, in relation to a number of factors involved in diabetes management, there appears to be an association with low SES. Evidence suggests that attending specialist diabetes services appears to be associated with better outcomes and individuals attending these services were of higher SES. Caution is needed, however, since two of the studies were cross sectional and hence causality could not be inferred. Nevertheless, a profile appears to be emerging of low SES individuals who are less likely to regularly attend specialist diabetes services, are less likely to be on intensive regimens, are less likely to monitor blood glucose levels, are more likely to have higher HbA1c levels, are more likely to suffer complications arising from diabetes, may not have attended structured education and are less knowledgeable about their diabetes care. The evidence about diabetes management has some limitations. First, there were few studies reporting on some aspects of diabetes management (access to diabetes care [31;43;51], SMBG [34;37;43] or the adoption of an IIR [21;43;46]) and second, very few of the studies reporting on diabetes management carried out adjusted analysis in order to identify SES as an independent predictor.

### Potential pathways involved in poorer outcome for low SES individuals

This review has identified poorer outcomes and poorer access to services for adults with lower SES and type 1 diabetes. However, the review did not identify research explaining why adults with lower SES have poorer outcomes and the pathways involved in lack of access to services. Although access to essential healthcare plays only a small part in differences in mortality, healthcare services may play an important role in lessening the impact of growing inequalities [7]. In their review, Brown et al. [4] utilise a conceptual framework, involving a complex web of interrelated influences, in order to explain the potential mechanisms whereby socioeconomic position may affect health outcomes amongst individuals with diabetes. Access to care is posited as one of the ways in which socioeconomic position may influence health outcomes. The authors suggest that poor health outcomes result from a combination of lack of access to high quality healthcare, resulting in inadequate and inferior treatment, and deficits in self-care behaviour [4].

In the current review, socioeconomic disparities were consistently found in studies regardless of the type of healthcare system adopted by the country. This highlights, in common with other reviews, that socioeconomic inequalities may persist even amongst individuals with access to a universal healthcare system [5;11] and there have been calls for research to explain why these individuals benefit less from access to services than their counterparts in high SES groups [5]. One quantitative survey conducted in Australia, targeting an area of deprivation, has explored barriers and enablers to healthcare access for young adults with type 1 diabetes [55]. Greater satisfaction with services was found to be associated with higher levels of income and education. Satisfaction with services was also associated with having recently had contact with specialist diabetes services, having a lower HbA1c, having an awareness of HbA1c result and having lower depression and anxiety scores. A previous unsatisfactory experience was found to be a barrier to further attendance with a number of participants reporting (in open ended responses) a feeling of being judged by overly critical staff. Facilitators of engagement with services included continuity of care, time of day for appointment and distance from home to the clinic. These responses were not reported by SES and although the target population comprised individuals from a deprived area, the study response rate was low (24%) and hence may not be representative of this group. The type 1 diabetes regimen demands enormous effort. In addition, factors involved in poorer outcomes for adults of low SES are likely to be multifaceted involving personal, social, financial and community factors [18]. It is therefore of utmost importance to ensure that access to diabetes healthcare is equitable, particularly for those who are most vulnerable in society, since it is known that the complications associated with poor diabetes management may be prevented through the adoption of effective regimens [3].

### Strengths and limitations

The review was limited by large variability in: designs used (cohort, cross sectional and case control studies); samples (centre-based versus population-based); age ranges and definitions of socioeconomic variables. This review was restricted to peer reviewed journal literature published in the English language so it is possible that relevant publications may have been excluded. Even with the above limitations, a consistent picture emerged of low SES associations with poorer outcomes in terms of mortality, morbidity and diabetes management. The results of this review in relation to morbidity and diabetes management resonate with other socioeconomic reviews [5;11;56]. Previous studies have found low SES was associated with indicators of poor diabetes management and worse intermediate and long-term outcomes [5;11]. Sawka et al. [56], in a systematic review of SES and hypoglycaemia, concluded that low SES was associated with increased incidence of severe hypoglycaemia. There was, however, little overlap in terms of studies included in these reviews and the current review due to the strict criterion of including only those studies that reported on adults with type 1 diabetes separately. The current review was strengthened by the large number of studies (27 of 33) that focused on type 1 diabetes alone and hence it avoided some of the potential problems arising from distinguishing adults with type 1 diabetes studies that also include adults with type 2 diabetes. This is the first review to report these disparities solely in relation to adults with type 1 diabetes.

## Conclusion

This review has identified that inequalities in mortality, morbidity and diabetes management were associated with socioeconomic factors for adults with type 1 diabetes even amongst patients with access to a universal healthcare system. Given the potential for diabetes management to mitigate the adverse effects of low socioeconomic status, further research is required to examine some of the potential causal pathways involved in the persistence of these inequalities.

## Supporting information

S1 Table Search strategy applied to Medline. (DOCX)

S2 Table Quality assessment form. (XLSX)

S3 Table PRISMA checklist. (DOC)

## References

[1] National Institute for Health and Care Excellence. Continuous subcutaneous insulin infusion for the treatment of diabetes mellitus. Technology Appraisal Guidance No 151. NICE 2008. http://www.nice.org.uk/guidance/ta151. Accessed 30 Aug 2016.

[2] International Diabetes Federation. *IDF Diabetes Atlas*. 7 ed Brussels, Belgium: International Diabetes Federation; 2015.

[3] DCCT Research Group. The effect of intensive treatment of diabetes on the development and progression of long-term complications in insulin-dependent diabetes mellitus. *New England Journal of Medicine* 1989;329:977–6. 10.1056/NEJM199309303291401 8366922

[4] Brown AF, Ettner SL, Piette J, Weinberger M, Gregg E, Shapiro MF, et al Socioeconomic position and health among persons with diabetes mellitus: a conceptual framework and review of the literature. *Epidemiologic Reviews* 2004;26:63–77. 10.1093/epirev/mxh002 15234948

[5] Grintsova O, Maier W, Mielck A. Inequalities in health care among patients with type 2 diabetes by individual socio-economic status (SES) and regional deprivation: a systematic literature review. *International Journal for Equity in Health* 2014;13:43 10.1186/1475-9276-13-43 24889694 PMC4055912

[6] Mackenbach JP. The persistence of health inequalities in modern welfare states: the explanation of a paradox. *Social Science & Medicine* 2012;75(4):761–9. 22475407 10.1016/j.socscimed.2012.02.031

[7] Whitehead M, Evandrou M, Haglund B, Diderichsen F. As the health divide widens in Sweden and Britain, what's happening to access to care? *BMJ* 1997;315((7114)):1006–9. 9365303 10.1136/bmj.315.7114.1006 PMC2127673

[8] Cortina S, Repaske DR, Hood KK. Sociodemographic and psychosocial factors associated with continuous subcutaneous insulin infusion in adolescents with type 1 diabetes. *Pediatric Diabetes* 2010;11(5):337–44. 10.1111/j.1399-5448.2009.00593.x 19761529

[9] Wood J, Lin M, Connor C, Ruedy K, Beck R, Kollman C, et al Race and socioeconomic status are associated with insulin pump therapy in youth in the first year following diagnosis of type 1 diabetes. *Diabetes Technology & Therapeutics* 2013;15:A25–A26. 10.1089/dia.2013.0132 PMC3817890 23869706

[10] Simmons JH, Chen V, Miller KM, McGill JB, Bergenstal RM, Goland RS, et al Differences in the management of type 1 diabetes among adults under excellent control compared with those under poor control in the T1D Exchange Clinic Registry. *Diabetes Care* 2013;36(11):3573–7. 10.2337/dc12-2643 24026543 PMC3816911

[11] Ricci-Cabello I, Ruiz-Perez I, Olry de Labry-Lima A, Marquez-Calderon S. Do social inequalities exist in terms of the prevention, diagnosis, treatment, control and monitoring of diabetes? A systematic review. *Health & Social Care in the Community* 2010;18(6):572–87. 21040063 10.1111/j.1365-2524.2010.00960.x

[12] Forssas E, Manderbacka K, Arffman M, Keskimäki I. Socio-economic predictors of mortality among diabetic people. *European Journal of Public Health* 2012;22(3):305–10. 10.1093/eurpub/ckr044 21498561

[13] Forssas E, Arffman M, Koskinen S, Reunanen A, Keskimäki I. Socioeconomic differences in mortality among diabetic people in Finland. *Scandinavian Journal of Public Health* 2010;38(7):691–8. 10.1177/1403494810376427 20651001

[14] Anderson SG, Malipatil NS, Roberts H, Dunn G, Heald AH. Socioeconomic deprivation independently predicts symptomatic painful diabetic neuropathy in type 1 diabetes. *Primary care diabetes* 2014;8(1):65–9. 10.1016/j.pcd.2013.08.004 24211151

[15] Butalia S, Johnson JA, Ghali WA, Rabi DM. Clinical and socio-demographic factors associated with diabetic ketoacidosis hospitalization in adults with Type 1 diabetes. *Diabet Med* 2013;30(5):567–73. 10.1111/dme.12127 23323955

[16] Nadas J, Putz Z, Fovenyi J, Gaal Z, Gyimesi A, Hidvegi T, et al Cardiometabolic risk and educational level in adult patients with type 1 diabetes. *Acta Diabetologica* 2009;46(2):159–62. 10.1007/s00592-008-0065-4 18843447

[17] Osan JK, Punch JD, Watson M, Chan YX, Barrie P, Fegan PG, et al Associations of demographic and behavioural factors with glycaemic control in young adults with type 1 diabetes mellitus. *Internal Medicine Journal* 2016;46(3):332–8. 10.1111/imj.12991 26748888

[18] Rawshani A, Svensson AM, Rosengren A, Eliasson B, Gudbjornsdottir S. Impact of Socioeconomic Status on Cardiovascular Disease and Mortality in 24,947 Individuals With Type 1 Diabetes. *Diabetes Care* 2015;38(8):1518–27. 10.2337/dc15-0145 25972573

[19] Sastre J, Pines PJ, Moreno J, Aguirre M, Blanco B, Calderon D, et al Metabolic control and treatment patterns in patients with type 1 diabetes in Castilla-La Mancha: the DIAbetes tipo 1 in Castilla La Mancha study. *Endocrinologia y Nutricion* 2012;59(9):539–46. 10.1016/j.endonu.2012.07.003 23039989

[20] Secrest AM, Costacou T, Gutelius B, Miller RG, Songer TJ, Orchard TJ. Association of socioeconomic status with mortality in type 1 diabetes: the Pittsburgh epidemiology of diabetes complications study. *Annals of Epidemiology* 2011;21(5):367–73. 10.1016/j.annepidem.2011.02.011 21458730 PMC3070912

[21] Secrest AM, Costacou T, Gutelius B, Miller RG, Songer TJ, Orchard TJ. Associations between socioeconomic status and major complications in type 1 diabetes: the Pittsburgh epidemiology of diabetes complication (EDC) Study. *Annals of Epidemiology* 2011;21(5):374–81. 10.1016/j.annepidem.2011.02.007 21458731 PMC3079455

[22] Weinstock RS, Xing D, Maahs DM, Michels A, Rickels MR, Peters AL, et al Severe hypoglycemia and diabetic ketoacidosis in adults with type 1 diabetes: results from the T1D Exchange clinic registry. *Journal of Clinical Endocrinology & Metabolism* 2013;98(8):3411–9. 23760624 10.1210/jc.2013-1589

[23] World Health Organisation. Health for the World's Adolescents: A Second Chance in the Second Decade. WHO 2014. http://www.who.int/maternal_child_adolescent/documents/second-decade/en/. Accessed 12 Feb 2017.

[24] HMSO. The Medicines for Human Use (Clinical Trials) Regulations 2004. The National Archives 2004. http://www.legislation.gov.uk/uksi/2004/1031/pdfs/uksi_20041031_en.pdf. Accessed 12 Feb 2017.

[25] HMSO. The Medicines for Human Use (Clinical Trials) Amendment (N0.2) Regulations 2006. The National Archives 2006. http://www.legislation.gov.uk/uksi/2006/2984/pdfs/uksi_20062984_en.pdf. Accessed 12 Feb 2017.

[26] HMSO. The Mental Capacity Act (2005). The National Archives 2005. http://www.legislation.gov.uk/ukpga/2005/9/pdfs/ukpga_20050009_en.pdf. Accessed 12 Feb 2017.

[27] HMSO. The Children Act 2004. The National Archives 2004. http://www.legislation.gov.uk/ukpga/2004/31/pdfs/ukpga_20040031_en.pdf. Accessed 12 Feb 2017.

[28] Booth A, Papaioannou D, Sutton A. Systematic Approaches to a Successful Literature Review. London: Sage; 2012.

[29] Critical Appraisal Skills Program (CASP). 12 Questions to help you make sense of cohort study. CASP 2013. http://www.casp-uk.net/. Accessed 30 Aug 2016.

[30] Moher D, Liberati A, Tetzlaff J, Altman DG, PRISMA Group. Preferred reporting items for systematic reviews and meta-analyses: the PRISMA Statement. *Open Medicine*: *A Peer-reviewed*, *Independent*, *Open-access Journal* 2009;3(3):e123–e130. PMC3090117 21603045

[31] Chaturvedi N, Stephenson JM, Fuller JH. The relationship between socioeconomic status and diabetes control and complications in the EURODIAB IDDM Complications Study. *Diabetes Care* 1996;19(5):423–30. 8732703 10.2337/diacare.19.5.423

[32] Forssas E, Keskimäki I, Reunanen A, Koskinen S. Widening socioeconomic mortality disparity among diabetic people in Finland. *European Journal of Public Health* 2003;13(1):38–43. 12678312 10.1093/eurpub/13.1.38

[33] Gnavi R, Petrelli A, Demaria M, Spadea T, Carta Q, Costa G. Mortality and educational level among diabetic and non-diabetic population in the Turin Longitudinal Study: a 9-year follow-up. *International Journal of Epidemiology* 2004;33(4):864–71. 10.1093/ije/dyh089 15131089

[34] Harris MI, Cowie CC, Howie LJ. Self-monitoring of blood glucose by adults with diabetes in the United States population. *Diabetes Care* 1993;16(8):1116–23. 8375241 10.2337/diacare.16.8.1116

[35] Hepburn DA, Langan SJ, Deary IJ, Macleod KM, Frier BM. Psychological and demographic correlates of glycaemic control in adult patients with type 1 diabetes. *Diabet Med* 1994;11(6):578–82. 7955976 10.1111/j.1464-5491.1994.tb02039.x

[36] Johansen K. Diabetes control and social class. *Practical Diabetes* 1986;3(2):93–5.

[37] Karter AJ, Ferrara A, Darbinian JA, Ackerson LM, Selby JV. Self-monitoring of blood glucose: language and financial barriers in a managed care population with diabetes. *Diabetes Care* 2000;23(4):477–83. 10857938 10.2337/diacare.23.4.477

[38] Leese GP, Wang J, Broomhall J, Kelly P, Marsden A, Morrison W, et al Frequency of severe hypoglycemia requiring emergency treatment in type 1 and type 2 diabetes: a population-based study of health service resource use. *Diabetes Care* 2003;26(4):1176–80. 12663593 10.2337/diacare.26.4.1176

[39] Lievre M, Marre M, Robert JJ, Charpentier G, Iannascoli F, Passa P, et al Cross-sectional study of care, socio-economic status and complications in young French patients with type 1 diabetes mellitus. *Diabetes & Metabolism* 2005;31(1):41–6. 15803112 10.1016/s1262-3636(07)70165-9

[40] Lloyd CE, Wing RR, Orchard TJ, Becker DJ. Psychosocial correlates of glycemic control: the Pittsburgh Epidemiology of Diabetes Complications (EDC) Study. *Diabetes Research & Clinical Practice* 1993;21(2–3):187–95. 8269821 10.1016/0168-8227(93)90068-g

[41] Matsushima M, Shimizu K, Maruyama M, Nishimura R, LaPorte RE, Tajima N. Socioeconomic and behavioural risk factors for mortality of individuals with IDDM in Japan: population-based case-control study. Diabetes Epidemiology Research International (DERI) US-Japan Mortality Study Group. *Diabetologia* 1996;39(6):710–16. 8781767 10.1007/BF00418543

[42] Mühlhauser I, Overmann H, Bender R, Bott U, Berger M. Risk factors of severe hypoglycaemia in adult patients with Type I diabetes: a prospective population based study. *Diabetologia* 1998;41(11):1274–82. 10.1007/s001250051065 9833933

[43] Mühlhauser I, Overmann H, Bender R, Bott U, Jörgens V, Trautner C, et al Social status and the quality of care for adult people with type I (insulin-dependent) diabetes mellitus: a population-based study. *Diabetologia* 1998;41(10):1139–50. 10.1007/s001250051043 9794099

[44] Mühlhauser I, Overmann H, Bender R, Jörgens V, Berger M. Predictors of mortality and end-stage diabetic complications in patients with Type 1 diabetes mellitus on intensified insulin therapy. *Diabet Med* 2000;17(10):727–34. 11110506 10.1046/j.1464-5491.2000.00372.x

[45] Pederson-Bjergaard U, Pramming S, Heller SR, Wallace TM, Rasmussen AK, Jorgensen HV, et al Severe hypoglycaemia in 1076 adult patients with type 1 diabetes: influence of risk markers and selection. *Diabetes-Metabolism Research and Reviews* 2004;20:479–86. 10.1002/dmrr.482 15386817

[46] Perros P, Deary IJ, Frier BM. Factors influencing preference of insulin regimen in people with type 1 (insulin-dependent) diabetes. *Diabetes Research & Clinical Practice* 1998;39(1):23–9. 9597371 10.1016/s0168-8227(97)00109-5

[47] Robinson N, Edouard L, Diehl A, Fuller JH. Social class and risk factors for vascular disease in diabetes. *Diabete et Metabolisme* 1984;10(4):245–9. 6391975

[48] Robinson N, Lloyd CE, Stevens LK. Social deprivation and mortality in adults with diabetes mellitus. *Diabet Med* 1998;15(3):205–12. 10.1002/(SICI)1096-9136(199803)15:3<205::AID-DIA519>3.0.CO;2-# 9545121

[49] Rossing P, Hougaard P, Borch-Johnsen K, Parving HH. Predictors of mortality in insulin dependent diabetes: 10 year observational follow up study. *BMJ* 1996 28;313(7060):779–84. 8842069 10.1136/bmj.313.7060.779 PMC2352213

[50] Unwin N, Binns D, Elliott K, Kelly WF. The relationships between cardiovascular risk factors and socio-economic status in people with diabetes. *Diabet Med* 1996;13(1):72–9. 10.1002/(SICI)1096-9136(199601)13:1<72::AID-DIA21>3.0.CO;2-T 8741816

[51] Zgibor JC, Songer TJ, Kelsey SF, Weissfeld J, Drash AL, Becker D, et al The association of diabetes specialist care with health care practices and glycemic control in patients with type 1 diabetes: a cross-sectional analysis from the Pittsburgh epidemiology of diabetes complications study. *Diabetes Care* 2000;23(4):472–6. 10857937 10.2337/diacare.23.4.472

[52] Carstairs V, Morris R. *Deprivation and Health in Scotland*. Aberdeen: Aberdeen University Press; 1991.

[53] Townsend P, Phillimore P, Beattie A. *Health and Deprivation*: *Inequality in the North*. London: Croom Helm; 1988.

[54] Adams AS, Mah C, Soumerai SB, Zhang F, Barton MB, Ross-Degnan D. Barriers to self-monitoring of blood glucose among adults with diabetes in an HMO: a cross sectional study. *BMC Health Services Research* 2003 19;3(1):6 10.1186/1472-6963-3-6 12659642 PMC153532

[55] Kibbey KJ, Speight J, Wong JL, Smith LA, Teede HJ. Diabetes care provision: barriers, enablers and service needs of young adults with Type 1 diabetes from a region of social disadvantage. *Diabet Med* 2013;30(7):878–84. 10.1111/dme.12227 23659590

[56] Sawka AM, Boulos P, Talib AS, Gafni A, Thabane L, Papaioannou A, et al Low socioeconomic status and increased risk of severe hypoglycemia in type 1 diabetes: a systematic literature review. *Canadian Journal of Diabetes* 2007;31(3):233–41.

