# Supplementary material for: Socioeconomic inequalities in mortality, morbidity and diabetes management for adults with type 1 diabetes: A systematic review
Source: PLoS One. 2017 May 10;12(5):e0177210. doi: 10.1371/journal.pone.0177210 (PMC5425027; doi:10.1371/journal.pone.0177210)
Supplement: S1 Table — (DOCX) [file pone.0177210.s001.docx]

# S1 File

# Table A: Search terms for Medline

1. Type 1 diabet*.mp.
2. Diabetes mellitus, type 1/or type 1 diabetes.mp.
3. Insulin dependent diabet*.mp
4. 1 or 2 or 3
5. Socio-economic.mp
6. Socioeconomic.mp
7. Social class*.mp
8. Social status.mp
9. Poverty/ or impoverished.mp
10. Inequit*.mp
11. Equity.mp or health services accessibility/
12. Access*.mp
13. Healthcare disparit*.mp
14. Health care disparit*.mp
15. Health status disparit*.mp
16. 5 or 6 or 7 or 8 or 9 or 10 or 11 or 12 or 13 or 14 or 15
17. 4 and 16
18. Limit 17 to (english language and humans)
19. Limit 18 to adults
